# Identification of drought responsive proteins and related proteomic QTLs in barley

**DOI:** 10.1093/jxb/erz075

**Published:** 2019-02-28

**Authors:** Paweł Rodziewicz, Klaudia Chmielewska, Aneta Sawikowska, Łukasz Marczak, Magdalena Łuczak, Paweł Bednarek, Krzysztof Mikołajczak, Piotr Ogrodowicz, Anetta Kuczyńska, Paweł Krajewski, Maciej Stobiecki

**Affiliations:** 1 Institute of Bioorganic Chemistry Polish Academy of Sciences, Noskowskiego 12/14, 61–704 Poznań, Poland; 2 Institute of Plant Genetics Polish Academy of Sciences, Strzeszyńska 34, 60–479 Poznań, Poland; 3 Department of Mathematical and Statistical Methods, Poznań University of Life Sciences, Wojska Polskiego 28, Poznań, Poland

**Keywords:** 2D electrophoresis, barley, cereals, drought response, large-scale proteomics, mapping population, mass spectrometry, proteomic quantitative trait loci (pQTL)

## Abstract

Drought is a major abiotic stress that negatively influences crop yield. Breeding strategies for improved drought resistance require an improved knowledge of plant drought responses. We therefore applied drought to barley recombinant inbred lines and their parental genotypes shortly before tillering. A large-scale proteomic analysis of leaf and root tissue revealed proteins that respond to drought in a genotype-specific manner. Of these, Rubisco activase in chloroplast, luminal binding protein in endoplasmic reticulum, phosphoglycerate mutase, glutathione S-transferase, heat shock proteins and enzymes involved in phenylpropanoid biosynthesis showed strong genotype×environment interactions. These data were subjected to genetic linkage analysis and the identification of proteomic QTLs that have potential value in marker-assisted breeding programs.

## Teaser

In barley, Rubisco activase, luminal binding protein, phosphoglycerate mutase, glutathione S-transferase, heat shock proteins, and enzymes involved in phenylpropanoid biosynthesis respond to drought in a genotype-specific manner.

## Introduction

Drought is one of the main abiotic factors that limits plant growth and crop productivity (Farooq *et al.*, 2009). It has a complex impact on plants involving a multitude of biochemical mechanisms (Bechtold, 2018). Prolonged drought conditions negatively affect germination (Blum *et al.*, 1980), seedling growth (Soltani *et al.*, 2006), tillering, and flowering (Barnabás *et al*., 2008), as well as grain-related traits (Dolferus *et al.*, 2011). Since cereals are a major source of food for humans and animals, this abiotic stress poses a serious threat to world food security (Reynolds and Tuberosa, 2008). However, further substantial improvements to drought resistance require a broader implementation of biotechnologically driven crop breeding strategies (Collard and Mackill, 2008). Also, elucidating the mechanisms of drought resistance in plants adapted to arid environments may provide new strategies for engineering drought tolerance in crop species (Bechtold, 2018; Zhang and Bartels, 2018).

Barley (*Hordeum vulgare* L.) is extensively utilized in plant science as a model for studying cereal biology due to its autogamous nature, short life cycle, ease of cultivation, adaptability to various environments, and relatively small diploid genome (~5 Gbp) with a low chromosome number (2*n*=14) (Saisho and Takeda, 2011). The sequencing of its genome has greatly advanced genetic studies on this species (Mayer *et al.*, 2012; Muñoz-Amatriaín *et al.*, 2015; Mascher *et al.*, 2017).

Progress in gene sequencing technology, mass spectrometry and bioinformatics has improved our understanding of complex interactions between genetic, proteomic, and metabolomic networks involved in perceiving and regulating responses to abiotic stresses in plants (Moreno-Risueno *et al.*, 2010; Urano *et al.*, 2010; Cramer *et al.*, 2011; Rodziewicz *et al.*, 2014). Many proteomic surveys have already been performed on cereal crops (including barley) to elucidate the mechanisms involved in neutralizing the adverse effects of drought (Salekdeh *et al.*, 2002; Caruso *et al.*, 2009; Wendelboe-Nelson and Morris, 2012; Ashoub *et al.*, 2013; Kausar *et al.*, 2013; Rollins *et al.*, 2013; Vítámvás *et al.*, 2015; Chmielewska *et al.*, 2016; Ghatak *et al.*, 2017). However, high variability of the proteome and the small number of genotypes usually analysed make it difficult to precisely differentiate proteins solely related to drought occurrence from those implicated in drought resistance. On the other hand, the amount of data resulting from large-scale proteomic studies enables application of multivariate statistics and therefore evaluation of proteome fluctuations in relation to genetic variation. One such approach is proteomic quantitative trait locus (pQTL) analysis, in which protein abundance is correlated with genetic polymorphism (Burstin *et al.*, 1994; Raorane *et al.*, 2015).

In this report, we describe a large-scale untargeted proteomic analysis of drought-induced leaf and root proteome alterations in barley recombinant inbred lines (RILs), and also two parental varieties with a different degree of drought tolerance, analysed previously with different approaches (Górny, 2001; Chmielewska *et al.*, 2016). The drought conditions were applied at the early stage of barley development, shortly before above-ground shoot formation (tillering). This process determines the number of spikes and plays a role in yield formation (Głąb *et al.*, 2015; Xie *et al.*, 2016). It was shown that drought has a huge impact on tillering, which generally leads to a reduction of the yield and biomass (Blum *et al.*, 1990; Jamieson *et al.*, 1995; El Hafid, 1998). To evaluate proteome changes of 102 barley genotypes, we developed a novel approach of gel-based proteomic analysis. We identified proteins that significantly changed their accumulation pattern in drought conditions, but also those whose accumulation profiles significantly fluctuated between tested genotypes. We next linked the identified proteins to the genetic map constructed for the studied RILs (Mikołajczak *et al.*, 2016, 2017). The results of the performed pQTL analysis directly demonstrated the relationship between genome and proteome variations among tested barley genotypes. This study is a part of a multilevel investigation of barley’s responses to drought and other research has concerned genetic linkage analysis, phenotypic variability, and primary and secondary metabolite profiling (Mikołajczak *et al.*, 2016, 2017; Piasecka *et al.*, 2017; Swarcewicz *et al.*, 2017).

## Materials and methods

### Plant material and drought treatment

The workflow of the experiments in this study is shown in Supplementary Fig. S1 at *JXB* online. A segregating population of 100 RILs of spring barley (*Hordeum vulgare* L.), and their parental genotypes—Maresi and Cam/B1//CI08887/CI05761 (further referred to as Cam/B1/CI)—were used for the experiments. Maresi is a German semi-dwarf cultivar and Cam/B1/CI is a Syrian breeding line adapted to water-limited conditions. Parental genotypes were chosen based on previous results on their tolerance to reduced water supply and nutrition (Górny, 2001; Krzemińska and Górny, 2003), and were described in detail by Mikołajczak *et al.* (2016). RILs were derived by the single seed descent (SSD) method in combination with *in vitro* culture in the Institute of Plant Genetics Polish Academy of Sciences. To ensure a high level of homozygosity, F_8_ plants were used for the analyses. Plants were grown as described by Mikołajczak *et al.* (2017). Briefly, pots containing 9 kg of loamy soil mixed with sand at a weight ratio of 7:2 were used for the experiments. Each genotype was grown in control and drought conditions under partially controlled greenhouse conditions. Soil moisture was established at pF 2.2 (15.8 kPa) for control and pF 3.2 (158.5 kPa) for drought. The amount of added fertilizer was calculated on the basis of soil tests. Drought treatment was achieved 16 d after sowing and began at the three-leaf stage, shortly before tillering (phase 13 in the BBCH scale). The drought conditions lasted for 10 d, which also ensured coverage of the tillering stage. In each pot, 10 plants were cultivated and when harvested were treated as one pooled biological replication. The plant material (leaves and roots) was collected after the drought period and was immediately frozen in liquid nitrogen. The samples were ground in liquid nitrogen precooled adaptors for 45 s at 30 Hz frequency using an MM400 ball mill (Retsch, Germany). Pulverized tissue was stored at −80 °C until further analysis.

### Protein extraction and 2D electrophoresis of the proteins

Proteins were extracted using the method described by Hurkman and Tanaka (1986) with certain modifications. The sample preparation procedure as well as 2D electrophoresis conditions used for separation are shown in Supplementary Table S1, and were also described in details previously (Chmielewska *et al.*, 2016). Plant material was extracted from two biological replications and two technical repetitions were prepared from each biological sample. Image Master 2D Platinum 7.0 software (IMP7, GE Healthcare, USA) was used for quantitative gel analysis. Protein spots’ relative volume (%vol.) was quantified. This parameter is relatively independent of sample loading and staining variation. In the case of roots, we could not proceed with 11 RILs due to insufficient material. For each genotype and tissue, four gels in control and four in drought conditions were analysed. The images of 2D electrophoretic gels were deposited in the Dryad Digital Repository (https://doi.org/10.5061/dryad.6st3v7r; Rodziewicz *et al.*, 2019).

### Gel alignment procedure

Because the alignment of a large number of gels in IMP7 software is not possible, we developed a two-step matching procedure allowing identification of spots in all gels. In the first step, gels obtained from control and drought-treated plants were aligned. Protein spots were matched and annotated separately within each genotype in IMP7. Subsequently, one annotated gel (for first replication under drought) was selected for each genotype as a reference and the set of 102 reference gels was aligned in IMP7. Then, the protein spot numbers on reference gels were used to link protein matching sets identified in the two steps of the analysis (see Supplementary Fig. S2). The %vol. for each measured protein spot from all replications was extracted to a joint result file.

### In-gel digestion and protein identification by mass spectrometry

Protein spots were excised from the gels and digested with trypsin following the protocol described by Shevchenko *et al.* (1996) with certain modifications. Samples were analysed using a matrix-assisted laser desorption/ionization (MALDI)–time of flight (TOF) or MALDI-TOF/TOF mass spectrometer, the Autoflex or UltrafleXtreme model (Bruker Daltonics, Germany). The protein identification was performed with two mass spectrometric methods: peptide map fingerprinting (PMF) or peptide sequencing with the MS/MS method. The sample preparation procedure and the conditions for mass spectrometric analysis as well as protein identification were described previously (Chmielewska *et al.*, 2016).

### Data analysis

The data were approximated to a normal distribution by transforming the values of the measured protein abundances by log_e_(1000×%vol.). The statistical analyses were conducted in Genstat 17 (VSN International, 2011) and R software (R Development Core Team, 2008). Analysis of variance (ANOVA) was applied to test the significance of the mean differences among RILs and parental genotypes, of the differences between protein accumulation profiles in drought and control conditions (drought effect), and to identify proteins with a different level of reaction to drought among tested RILs (genotype×environment (G×E) interaction). The significant effects were selected at *P*<0.05 using Bonferroni correction (*P*<0.05/*z*, where *z* is the number of observed proteins: 0.05/257=0.000195 for leaves, and 0.05/381=0.00013 for roots). The chi-square test was used to identify significant deviations from homogeneous distributions in protein functional categories. Hierarchical cluster analysis was performed using heatmap2 in R on drought effects for proteins with significant G×E interaction with a minimum of 60 observations and was based on the Euclidean distance and complete linkage algorithm, with the missing data replaced by mean values; homogeneous groups of proteins were found using pvclust in R (with α=0.95). Principal coordinate analysis was performed for all proteins and also for functional categories. Correlation structure of proteins observed in at least 60 lines was analysed on the basis of Pearson correlation coefficients. Correlation networks were constructed separately for proteins observed in control and in drought by the WGCNA package in R (Langfelder and Horvath, 2008), and visualized by Cytoscape (Shannon *et al.*, 2003). The modules of correlated proteins were detected by clustering the topological overlay matrix (TOM) using the dynamic tree cut algorithm. Correlation coefficients were computed for all proteomic pairs and the Mann–Whitney test was applied to test the significance of the differences in correlation coefficients.

### Genotyping and pQTL analysis

Genotyping of parental forms and RILs was performed as described by Mikołajczak *et al.* (2016). QTL localization was performed for proteomic traits observed in at least 60 RILs, for each trait independently, with mixed linear model-based interval linkage mapping (Malosetti *et al.*, 2013). The computations were done in Genstat 17 using the compound symmetry model of environmental co-variation between control and drought conditions, genome-wide error rate smaller than 0.05, and selection of significant QTL effects in the final model at *P*<0.05. For each QTL, the two-LOD support interval was computed according to Xu and Hu (2010); mean length of all intervals, *m=*1.8 cM, was further used as a parameter in QTL annotation. Single nucleotide polymorphism (SNP) sequences taken from Close *et al.* (2009) were mapped to barley genome space in Ensembl Plants (v. 082214v1). The QTL intervals of length 2*m* around the closest markers were projected onto the genomic sequence, with the use of SNPs corresponding to the interval boundaries. Structural genes of corresponding proteins were considered at a distance smaller than 20 kbp from the pQTLs.

## Results

**Fig. 1.**
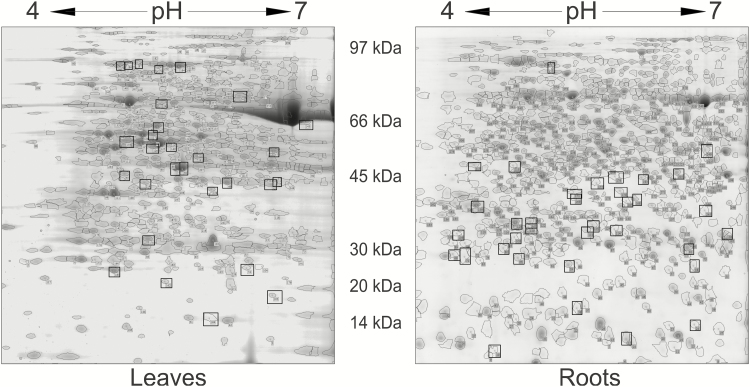
Examples of two-dimensional electrophoretic gels obtained during analysis of protein extracts from barley leaves (Maresi) and roots (Cam/B1/CI) with marked protein spots subjected for statistical analysis and identification. The numbering of the proteins on the gels corresponds to Supplementary Table S2 (leaves) and Supplementary Table S3 (roots). Proteins discussed in the text are additionally marked with rectangles.

The gel alignment procedure enabled the identification of 550 protein matchsets in leaves and 825 in roots observed with various frequencies, in 3–102 genotypes. The matchsets containing proteins detected in a minimum of 20 genotypes and with correlation between technical replicates *R*>0.25 were selected for statistical analysis, which provided 257 and 381 protein sets in leaves and roots, respectively (Fig. 1). From these, we were able to identify 184 proteins in leaves and 231 proteins in roots with mass spectrometry (see Supplementary Tables S2 and S3, respectively). The gel electrophoretic 2D separations allowed reproducible detection of protein spots between replicates. Examples of proteins spots in control and drought conditions are presented in Fig. 3. The numbering of the proteins in the text corresponds to the numbering in Supplementary Table S2 (leaves) and Supplementary Table S3 (roots). The identified proteins were grouped based on their putative function in biological processes (Fig. 3). The described quantitative changes of the proteins concern single protein isoforms identified under defined spot detected on the 2D gel.

**Fig. 2.**
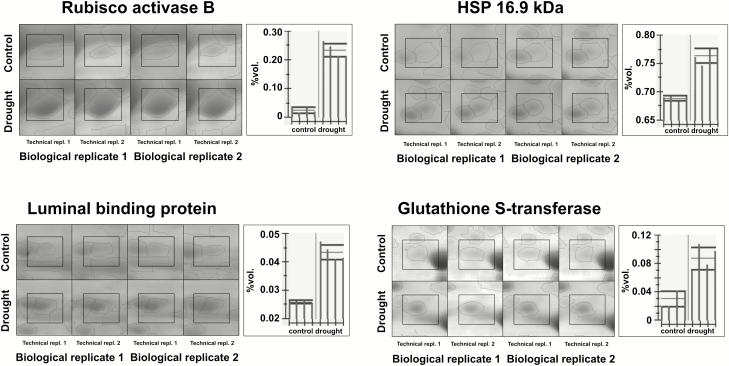
Gel fragments showing reproducibility between biological replicates and technical replications. The examples represent the accumulation pattern of selected proteins in control and drought conditions in leaves: Rubisco activase B (no. 140) and luminal binding protein (no. 86); and in roots: heat shock protein 16.9 kDa (no. 125), and glutathione S-transferase (no. 114). The vertical bars represent intensities of spots in four control and four drought treated samples; horizontal lines represent mean value ± standard deviation.

**Fig. 3.**
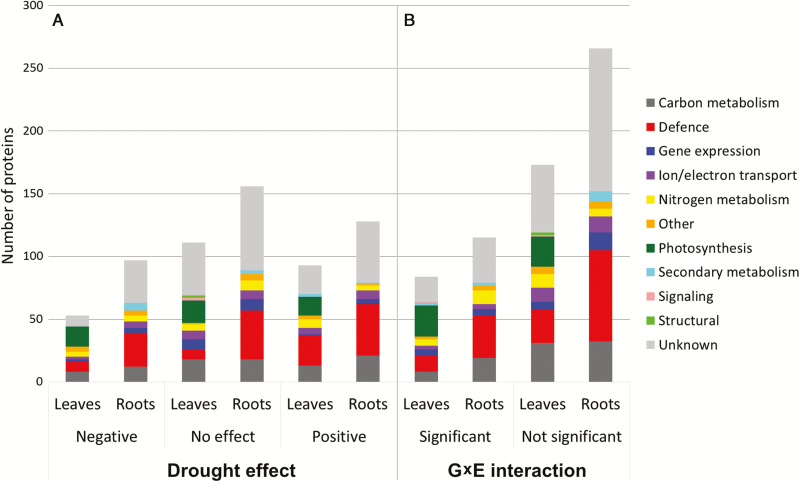
Functional classification of proteins identified in leaves and roots in RILs according to the displayed drought effect (A) and genotype×environment (G×E) interaction (B).

### The changes of drought-induced protein accumulation in parental genotypes

**Fig. 4.**
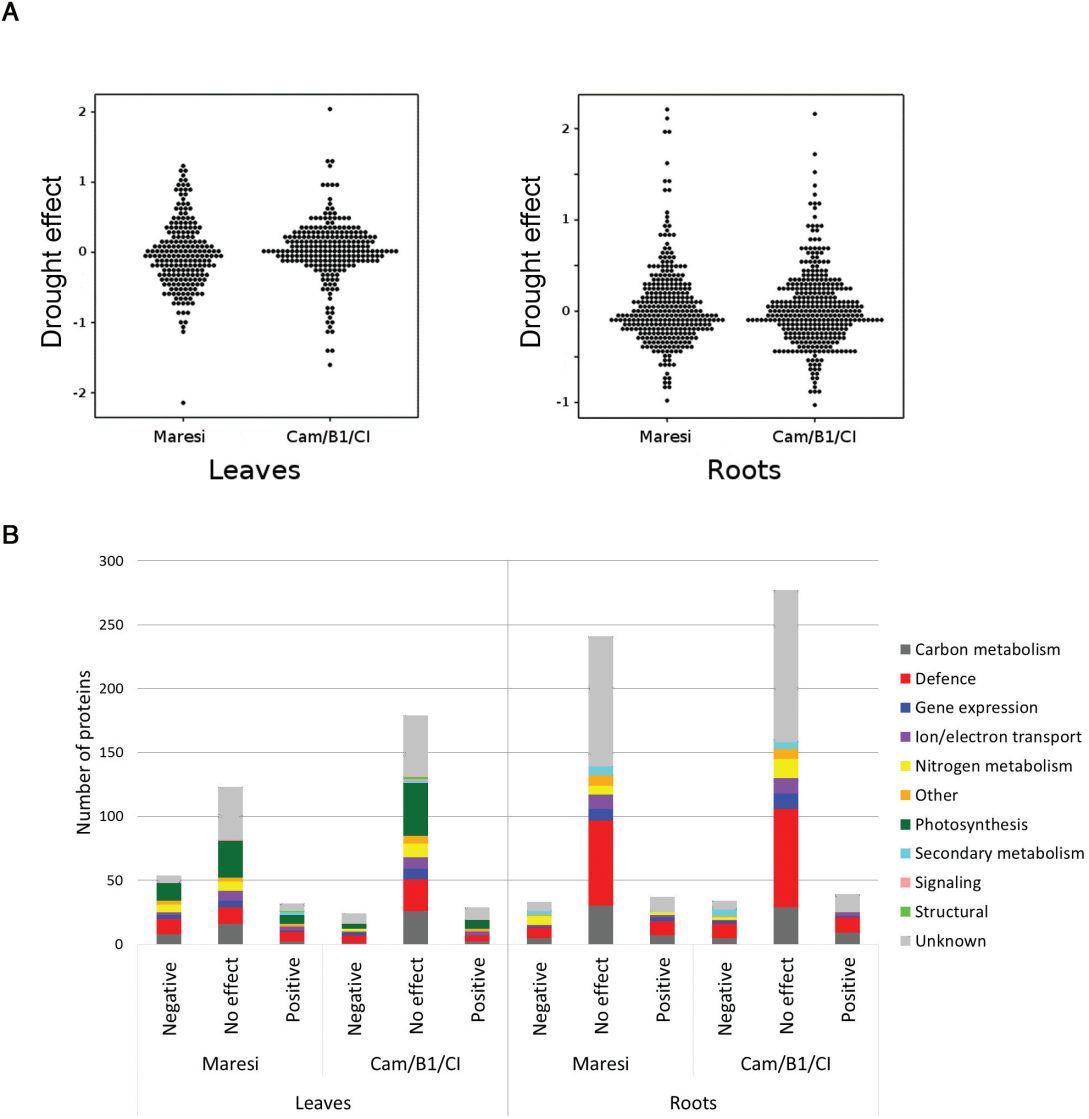
Distribution of drought effects in Maresi and Cam/B1/CI for all proteins (A) and with regard to functional classification (B).

In leaf tissue, the accumulation profiles of 209 proteins in Maresi and 232 in Cam/B1/CI were monitored and 198 of them were matched in both genotypes (Supplementary Table S4). In roots, 296 proteins were common for both genotypes out of a total 311 and 350 observed in Maresi and Cam/B1/CI, respectively (Supplementary Table S5). In leaf tissue, a similar number of proteins exhibited a positive drought effect in both genotypes. However, in Maresi the negative drought effect was more pronounced, which in Cam/B1/CI shifted into no drought effect (Fig 4A; Supplementary Table S4). The most striking differences in the accumulation profiles between parental genotypes were observed in the categories photosynthesis, defense mechanisms and carbon metabolism (Fig. 4B; Supplementary Table S6). The distribution of drought effects in root tissue was very similar for both genotypes, apart from nitrogen metabolism. In this category, more proteins with decreased accumulation were found in Maresi, whereas in Cam/B1/CI, mainly no reaction to drought was observed (Fig. 4B; Supplementary Table S6).

### The changes of drought-induced protein accumulation in RILs

**Fig. 5.**
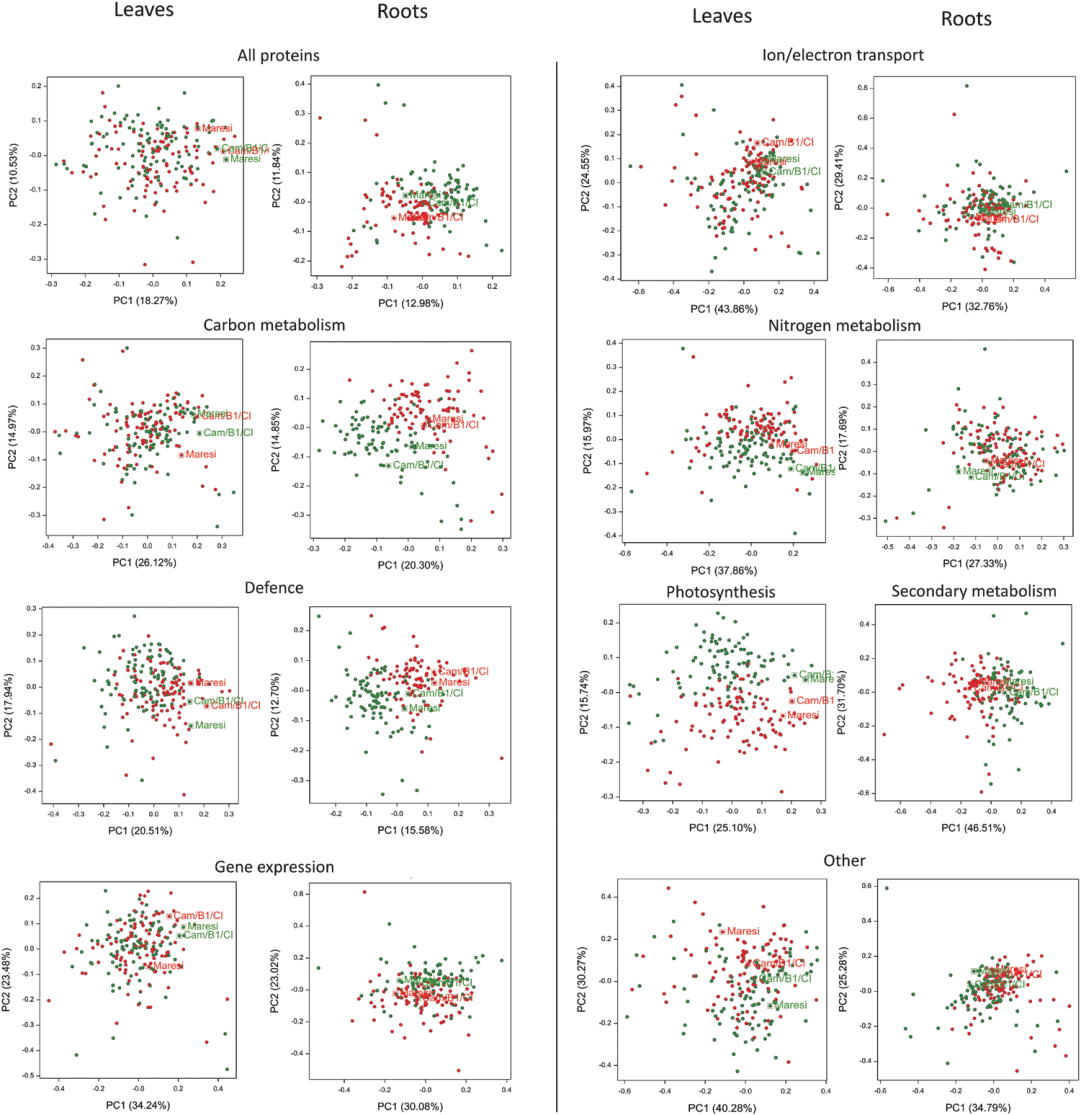
Principal coordinate analysis of RILs and parental genotypes for all observed proteins and proteins in functional groups. Green circles, control samples; red circles, drought samples; circles in squares mark parental forms.

The mean values of protein relative abundance were significantly different among RILs for all 257 leaf proteins and 378 out of 381 root proteins (*P*<0.05, Bonferroni correction for multiple testing) (see Supplementary Tables S2, S3). The fractions of drought-responsive proteins were similar in both plant tissues—57% (146 proteins) in leaves and 59% (225) in roots, of which 64% (93) and 57% (128) increased their abundances in drought, respectively (Supplementary Tables S2, S3). In leaf tissue, the distribution of proteins with negative, positive, and without drought effect was similar in all functional groups except for defense mechanisms, where the number of proteins with positive drought effect was significantly higher (χ^2^ test, *P*=0.003). No differences in drought effect distribution were observed in roots (χ^2^ test, *P*=0.390). The differentiation between protein levels in functional categories observed in control and drought conditions was more pronounced in root tissue, apart from nitrogen metabolism, where no clear separation was observed. In leaves, the differentiation was registered only in the categories photosynthesis, nitrogen metabolism, and defense mechanisms (Fig. 5). Proteins that exhibited high mean drought effects (positive or negative; below the 5th or above the 95th percentile of the distribution effects) were present abundantly in categories photosynthesis (11 proteins), defense mechanisms (five proteins in leaves, 13 proteins in roots), and carbon metabolism (just one protein in leaves, but 10 proteins in roots). In leaves, the highest positive mean drought effects were found for ribulose bisphosphate carboxylase/oxygenase activase B (Rubisco activase B) (no. 140), two isoforms of luminal binding protein (BiP) (nos 86 and 87), and HSP 70 kDa (no. 76) (Supplementary Table S2). The most negative drought effects were observed for two isoforms of Rubisco activase A (nos 126 and 128) and ferredoxin-NADP^+^ reductase (nos 52 and 55) (Supplementary Table S2). In root tissue, the highest positive mean drought effects were observed for two isoforms of HSP 16.9 kDa (nos 123 and 125), P-loop containing NTPase (no. 165), 2,3-bisphosphoglycerate-independent phosphoglycerate mutase (iPGM) (no. 10), NADP-dependent malic enzyme (no. 159), *S*-adenosylmethionine synthase 1 (no. 193), and cold regulated protein (no. 69) (Supplementary Table S3). The highest mean negative drought effects were noted for enzymes related to secondary metabolism: phenylalanine ammonia-lyase (PAL) (nos 168, 169 and 170), caffeic acid *O*-methyltransferase (no. 59); antioxidant enzymes: mitochondrial superoxide dismutase (no. 205), ascorbate peroxidase (no. 38); and 26S protease regulatory subunit 4 homolog (no. 12), 14-3-3-like protein A (no. 1), fructokinase (no. 100), germin-like protein 8-5 (no. 106) and tubulin α-2 chain (no. 217) (Supplementary Table S3).

### Genotype×environment interactions and correlation analysis

**Fig. 6.**
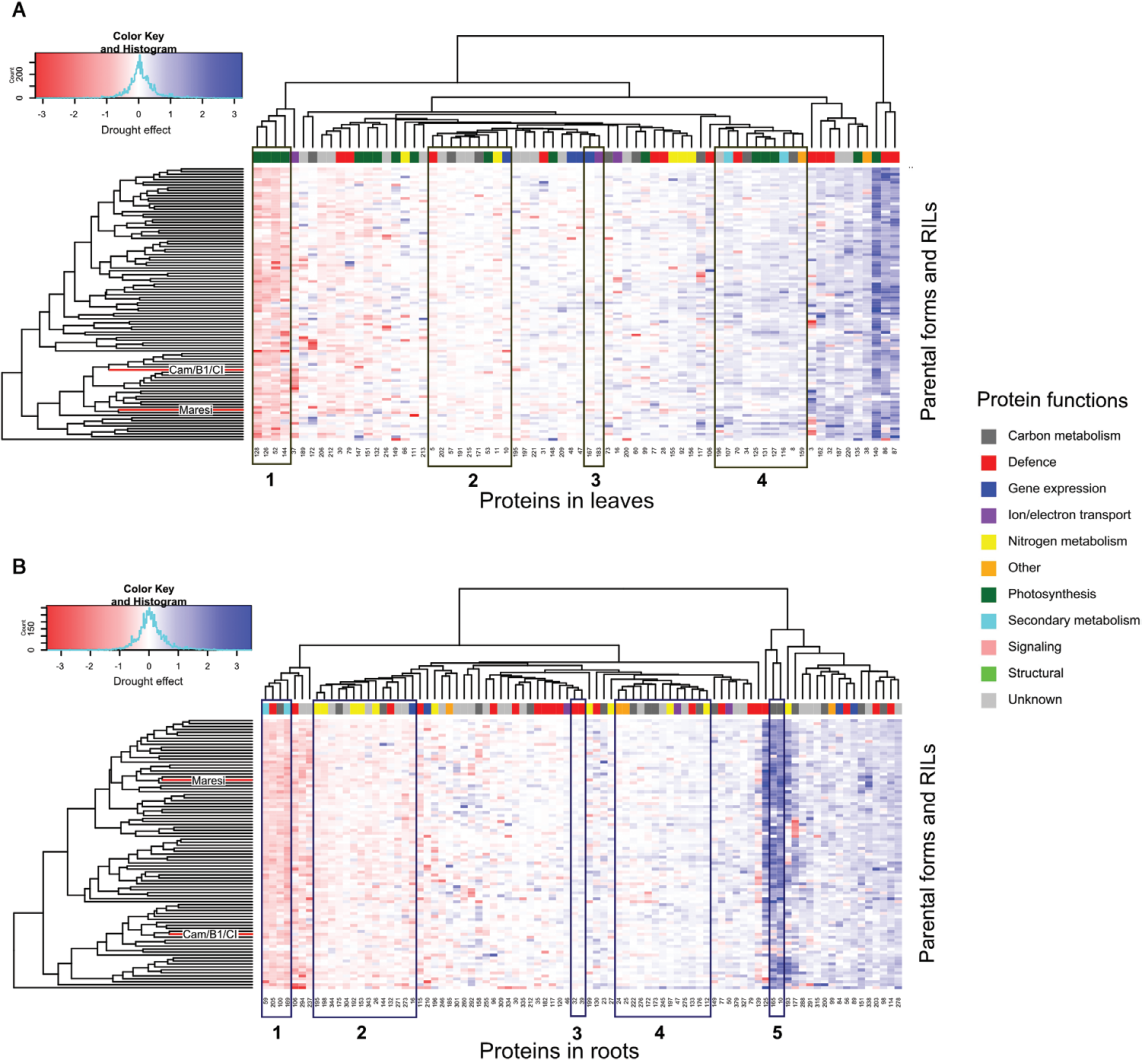
Hierarchical clustering analysis visualization of drought effects for leaf (A) and root (B) proteins exhibiting significant G×E interaction with minimum of 60 observations in all tested genotypes. Protein clusters for which a statistically similar reaction to drought was noted are marked by boxes. Parental lines are marked with red branches.

The G×E interactions were found to be significant for 84 proteins in leaves and 115 proteins in roots, which, in both cases, accounted for about 30% of all analysed proteins (Fig. 3B; Supplementary Tables S2, S3). Among them, 55 leaf proteins and 82 root proteins also exhibited a significant drought effect. The strongest G×E interactions were found for proteins that also showed high mean drought effect, i.e. leaf proteins (nos 86, 87, 126, 128, and 140), and root proteins (nos 10, 125, and 165), glutathione S-transferase (GST) (no. 114) and phosphoglycerate mutase (dPGM) (no. 177) (see Supplementary Tables S2, S3). The significant G×E interactions in leaves were more frequently found for proteins related to photosynthesis than other categories (χ^2^ test, *P*=0.049). Among proteins exhibiting significant interaction effects, four and five clusters of proteins with similar reactions were found in leaves and roots, respectively (Fig. 6).

**Fig. 7.**
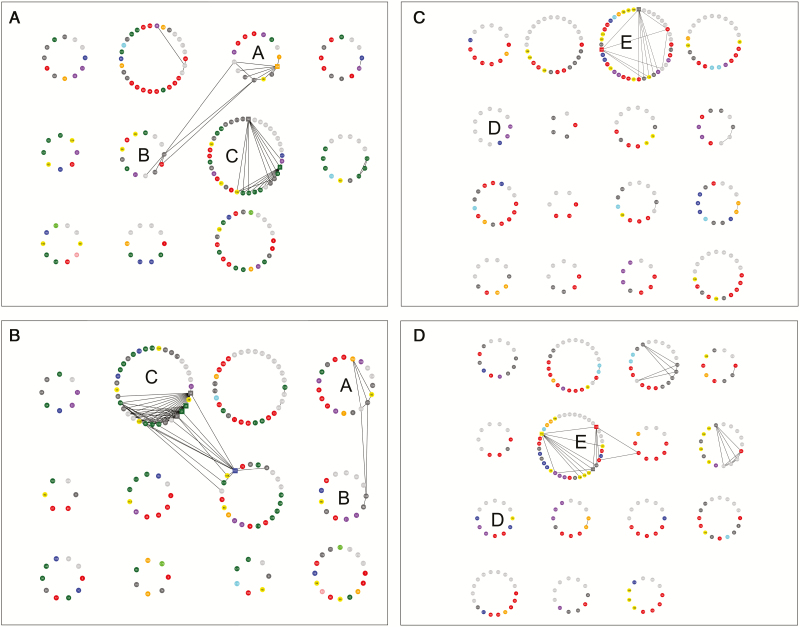
Correlation networks constructed for proteins observed in leaves in control (A) and drought conditions (B) and roots in control (C) and drought conditions (D). Proteins are represented by circles with numbers. Circle colors correspond to groups of proteins with different functions (for legend see Fig. 6). Edges link correlated proteins. Correlation matrix was transformed to the TOM, and only edges corresponding to elements of TOM greater than 0.15 are drawn. Squares represent ‘hubs’, i.e. proteins with the largest numbers of edges. Proteins are grouped into modules, i.e. groups of mostly correlated proteins, found by clustering based on the TOM and the dynamic tree cut algorithm. The modules with a large number of proteins (>60%) occurring in both control and drought are marked with letters (A)–(E).

**Fig. 8.**
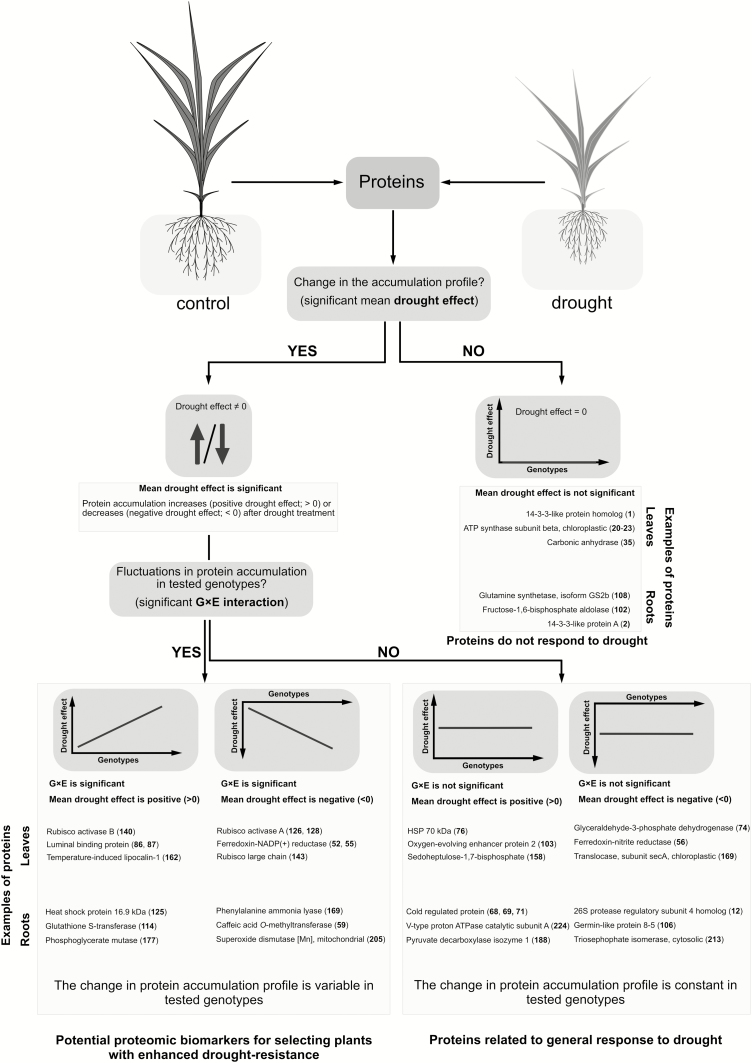
Model of drought response showing differences in protein accumulation profile in barley mapping population. Among drought-responsive proteins (positive or negative drought effect) one can distinguish those in which the accumulation profile changed depending on tested genotype (significant G×E interaction), and those in which the change in the accumulation profile was constant in tested genotypes. Due to variable level of accumulation between tested genotypes, proteins with significant G×E interaction may serve as potential biomarkers for selecting plants with higher degree of drought resistance. The numbers in bold with the protein names refer to proteins in Supplementary Table S2 or S3.

The sets of most correlated proteins (modules) defined in the correlation networks consisted of proteins with various functions (Fig. 7). The largest number of correlations in leaf tissue was found for ferredoxin-NADP^+^ reductase (nos. 49, 51), fructose-1,6-bisphosphate aldolase (nos 57 and 58), thiamine pyrophosphate (TPP)-dependent protein (no. 164), and translational elongation factor Tu (no. 167); and in root tissue for HSP 17.6 kDa (no. 128), phosphoglycerate mutase (no. 176), and glutamine synthetase (no. 112) (Fig. 7). Several subsets of proteins preserved their correlation structure between control and drought conditions. For example, in leaves, these proteins appeared in modules (a), (b), and (c), and together contained 13 (33% of all) carbon metabolism proteins and four (10%) defense proteins. The similar sets of root proteins in both conditions appeared in modules (d) and (e), and included seven (30%) nitrogen metabolism proteins and four (4%) defense proteins (Fig. 7). The structure of protein accumulation was more positively correlated in leaves than in roots. In leaf tissue, 73% and 77% of correlations were positive in control and drought conditions, respectively, and the shift of the distribution of correlations towards more positive values in drought was statistically significant (Mann–Whitney *U*-test, *P*<0.001). In roots, 59% and 58% of correlations were positive in control and drought conditions, respectively, and the distribution of correlations in both conditions was statistically similar (Mann–Whitney *U*-test, *P*=0.059). The model of drought response in tested barley genotypes with regard to drought effect and G×E interaction is presented in Fig. 8.

### Proteomic QTL analysis

The localization and statistical characteristics of detected pQTLs are presented in Supplementary Tables S7 and S8 for leaf and root tissue, respectively. In total, 31 pQTLs for leaves and 48 pQTLs for roots were mapped and distributed among almost all linkage groups, except from 1H.1 in leaves and 5H.2 in both tissues. Proteomic QTLs were found in particular in 3H.1 (both tissues) and 5H.3 (roots) linkage groups. In both tissues and conditions, the number of pQTLs for which the Maresi allele increased the protein level (negative additive effect) was larger than the number of pQTLs for which the Cam/B1/CI allele increased the protein level (positive additive effect) (see Supplementary Tables S7, S8). Several pQTLs co-localized with QTLs for yield-related traits identified previously (Table 1) (Mikołajczak *et al.*, 2016, 2017).

**Table 1.** List of proteins for which pQTL was co-localized with yield-related traits identified in the genetic linkage analysis^*a*^

Protein^*b*^	Yield related traits	Marker	Linkage group
Betaine aldehyde dehydrogenase (no. 58, roots)	1000-grain weight, grain weight per main spike	SNP 6655-978	1H.1
Thioredoxin O (no. 165, leaves)	Length of main spike	SNP 3886-313	3H
Unknown protein (no. 191, leaves)	1000-grain weight, heading stage, length of main stem	SNP ABC07496-pHv1343-02	3H.1
Unknown protein (no. 214, leaves)	Length of main and lateral spikes, number of spikelets per main and lateral spike, grain weight per main spike, number of grains per lateral spike	SNP 3688-1291	3H.1
ATP synthase subunit β (no. 24, leaves)	Length of main stem and main spike, number of grains, grain weight per lateral spike, heading stage	SNP 2129-1928	4H
Glutamine synthetase (no. 109, roots) HSP 70 kDa (no. 78, leaves)	Number and weight of grains per main spike	SNP 2055-947	4H
Phenylalanine ammonia lyase (no. 171, roots) Translocase subunit secA (no. 169, leaves) Unknown protein (no. 245, roots)	Length of main stem, grain weight per main spike	SNP 10207-1024	5H.1
Δ^1^-pyrroline-5-carboxylate synthetase (no. 81, roots)	Number of grains per main spike, number of spikelets per lateral spike, grain weight per plant and heading stage	SNP 314-559	5H.3
Luminal binding protein (no. 86, leaves) Unknown protein (no. 249, roots)	Grain weight per plant, heading stage	SNP ABC09320-1-1-112	7H.2

^*a*^ Based on the data from genetic linkage analysis performed by Mikołajczak *et al.* (2016, 2017).

^*b*^ The numbers with the protein names refer to Supplementary Tables S2, S3, where proteins identified in leaves and roots are listed.

The significant QTL×environment (control/drought) interactions were found for 22 leaf and 25 root pQTLs. The most significant pQTLs were detected for ATP synthase subunit beta (no. 24) in leaves and PAL (no. 171) in roots, both with stable effects across environmental conditions (see Supplementary Tables S7, S8). The largest number of pQTLs was found for proteins related to defense mechanisms (eight in leaves, 12 in roots). In this category, the increasing level of proteins was mostly determined by the Maresi allele, but a positive effect of the Cam/B1/CI allele was observed for thioredoxin (no. 165) in leaves; and for GST (no. 114) and GroEL-like type chaperonin (no. 120) in roots. Significant pQTL effects were also found for proteins exhibiting G×E interaction and high mean drought effect, i.e. BiP (no. 86) in leaves and GST (no. 114) and dPGM (no. 177) in roots (Supplementary Tables S7, S8). By classifying all QTL effects (in leaves and roots, control and drought—158 effects in total), in most cases the allele inherited from parental genotype with significantly larger protein accumulation in a given condition also increased the protein level in progeny. Seven pQTLs were located in close vicinity to the corresponding coding sequences. In leaves, these were found for actin (fragment) (no. 7; MLOC_54382), fructose-bisphosphate aldolase (nos 57 and 58; MLOC_39148), HSP 70 kDa (no. 78; MLOC_50972, MLOC_72334), cysteine synthase (no. 40; MLOC_22001), and triosephosphate isomerase (no. 170), and in roots for actin (no. 24; MLOC_71657) (Supplementary Tables S7, S8).

## Discussion

### Comparison of the results from untargeted proteomic approach to the previous studies

This large-scale comparative proteomic analysis required a completely different analytical approach from the one usually applied when studying only a small number of genotypes. To our knowledge, this is the first study describing proteome changes under drought conditions for such a large population of genotypes. Since the software for 2D gel analysis could not deal with this large dataset, the elaboration of a new method of gel analysis was necessary. The parental genotypes used in this study were earlier investigated under drought conditions with a classic gel-analysis approach (Chmielewska *et al.*, 2016). The developed gel alignment procedure together with application of multivariate statistical methods enabled us to identify proteome variations consistent with those reported earlier in Maresi and Cam/B1/CI (Chmielewska *et al.*, 2016). For instance, the differences in protein accumulation profiles between parental genotypes were highlighted more in drought than in control conditions, and with greater alterations in leaves (Fig. 4; Supplementary Tables S4–S6). The results from untargeted proteomic analysis confirmed a smaller drought reaction in Cam/B1/CI leaves with a higher ratio of proteins with an increased accumulation and also a larger decrease in protein content in Maresi (Fig. 4; Supplementary Table S6). Additionally, the distribution of drought-induced increases and decreases of the protein levels in the defined functional categories was similar to the previous results, especially for proteins related to defense mechanisms, carbon metabolism, nitrogen metabolism, and secondary metabolism (Fig. 4). Consistent with the data reported previously, in roots the reaction to drought was more conserved (Supplementary Table S6). The putative functions of the proteins identified in this study were thoroughly described in our earlier report with an indication that the activation of the mechanisms involved in the removal of reactive oxygen species (ROS) and enzymes involved in the biosynthesis of osmolytes influence the level of drought resistance (Chmielewska *et al.*, 2016).

Compared with other proteomic research conducted on barley subjected to drought, we also observed extensive drought-induced alterations in an accumulation of proteins involved in photosynthesis, defense mechanisms, carbon, nitrogen, and secondary metabolism (Fig. 5; Supplementary Tables S2, S3; Ford *et al.*, 2011; Wendelboe-Nelson and Morris, 2012; Ashoub *et al.*, 2013; Kausar *et al.*, 2013; Chmielewska *et al.*, 2016). The evaluation of the identified protein changes between various genotypes with a different degree of drought tolerance provides an insight into plant stress adaptation processes (Kosová *et al.*, 2011). However, in the small-scale studies, almost all of the identified drought-responsive proteins usually differentiate the studied genotypes, which, in turn, may lead to some inconsistencies when interpreting the results. For example, Wendelboe-Nelson and Morris (2012) reported decreased levels of photosynthetic enzymes in drought tolerant genotypes, whereas Kausar *et al*. (2013) found many of them at increased levels in the resistant variety and decreased in the sensitive one. Also, in our previous study, small and large subunits of Rubisco were identified as genotype differentiating proteins (Chmielewska *et al.*, 2016). In this study, however, most of the drought affected proteins did not show any significant differences between tested genotypes, including Rubisco (Fig. 3B; Supplementary Tables S2, S3), for which a decrease was recently assigned as a general symptom of declining photosynthesis and the protein itself may serve as a reservoir of nitrogen during adverse environmental conditions (Demirevska *et al.*, 2008; Aranjuelo *et al.*, 2011). The discrepancies between published results, especially regarding proteins involved in central metabolism, limit the discovery of proteins that may significantly influence drought resistance. However, a general conclusion can be drawn that higher constitutive and drought-induced accumulation of defense-related proteins, including ROS regulation, osmolytes biosynthesis, protein synthesis, folding and degradation, is more frequently observed in drought-tolerant genotypes (Ford *et al.*, 2011; Wendelboe-Nelson and Morris, 2012; Ashoub *et al.*, 2013; Kausar *et al.*, 2013; Rollins *et al.*, 2013; Chmielewska *et al.*, 2016). For example, mean accumulation of the pyrroline-5-carboxylate synthase (P5CS) (no. 81) increased after drought treatment in roots in the studied RILs, whereas an enzyme involved in proline degradation, Δ^1^-pyrroline-5-carboxylate dehydrogenase (no. 80) was unaffected (see Supplementary Table S3). In our previous studies, the constitutive levels of P5CS and proline were found higher in Cam/B1/CI, which due to its origin is likely more adapted to dry environments. In Maresi, on the other hand, the accumulation of P5CS was decreased in drought conditions and, in control samples, proline content was lower than in Cam/B1/CI (Chmielewska *et al.*, 2016; Swarcewicz *et al.*, 2017). In both tissues, the correlation structure of the proteins implicated in defense mechanisms was more affected by drought conditions than, for example, the structure of proteins related to carbon metabolism in leaves or nitrogen metabolism in roots (Fig. 7). It was also reflected in a significantly higher fraction of defensive proteins with positive drought effects in leaf tissue (Figs 3B, 4A), which may be linked to the elevated levels of ROS, which could arise due to excessive energy reaching the photosynthetic apparatus (Das and Roychoudhury, 2014). This included various isoforms of heat shock proteins, ascorbate peroxidases, superoxide dismutases, GSTs, metalloproteases, and also other proteins involved in ROS management (Supplementary Table S2).

### Large scale proteomic studies enable identification of the proteins involved in genotype-specific reaction to drought

In contrast to the studies performed on a small number of genotypes, our large-scale proteomic analysis enabled identification of the proteins exhibiting statistically significant differences in accumulation profile among studied RILs and conditions (Supplementary Tables S2, S3). The G×E interactions were found in all functional categories, but especially in photosynthesis (Fig. 3B; Supplementary Tables S2, S3). Rubisco activase is a chaperon protein involved in maintaining high photosynthetic rates under unfavorable environmental conditions and was suggested as a potential target for improving crop productivity (Kurek *et al.*, 2007; Lawson *et al.*, 2014). The second role of that protein was associated with protecting thylakoid-associated translation machinery and assisting in targeting ribosome complexes to the thylakoid membrane (Rokka *et al.*, 2001). The correlation structure between Rubisco activase A and B isoforms identified in this study was preserved in both environmental conditions, which may indicate common regulatory mechanisms during that stress (Fig. 7). The activity of Rubisco activase A was found to be sensitive to chloroplast redox potential whereas that of the smaller B isoform was not, which, to some extent, may explain the differences in accumulation pattern between the isoforms observed in this study (Fig. 6A; Supplementary Table S2; Zhang and Portis, 1999; Salvucci *et al.*, 2003). Also, during heat stress or water deficit, the diminished activity and accumulation pattern of Rubisco activase A was linked with a decline in the activation state of Rubisco, which ultimately led to decreased assimilation of photosynthetic CO_2_ (Carmo-Silva and Salvucci, 2011; Rollins *et al.*, 2013; Scafaro *et al.*, 2016; Perdomo *et al.*, 2017). On the other hand, the content of Rubisco activase B was reported to increase during heat, drought, or a combination of both conditions, also at the transcript level (Salekdeh *et al.*, 2002; Rollins *et al.*, 2013; Wang *et al.*, 2014; Tatli *et al.*, 2017). Luminal binding proteins play a major role in the activity of the unfolded protein response (Malhotra and Kaufman, 2007). The rapid increase in protein synthesis during abiotic stresses may lead to ‘overloading’ of endoplasmic reticulum, which in turn results in a higher number of misfolded and non-functional proteins (Valente *et al.*, 2009). The activation of the unfolded protein response helps to re-establish homeostasis in endoplasmic reticulum by restraining gene transcription and translation, and also by increasing the accumulation of chaperones assisting in protein folding (Ma and Hendershot, 2004). For example, overexpression of BiP in tobacco and soy plants resulted in increased drought resistance, but the changes in the expression level of drought-regulated genes, accumulation of osmolytes, or a decline in photosynthesis was not observed in those plants, which may suggest a different mechanism of counteracting negative drought effects (Alvim *et al.*, 2001; Valente *et al.*, 2009). Interestingly, the accumulation pattern of both BiP isoforms identified in leaves was similar to that of Rubisco activase B in tested RILs (Fig. 6A). In root tissue, high positive drought effects and strong G×E interactions were observed for various HSP 16.9 kDa isoforms (Supplementary Table S3). Drought-induced increase in accumulation of low molecular mass HSPs was already associated with maintaining the stability and integrity of cellular architecture, which may, for example, positively influence the plant’s ability to recover after rewatering (Waters *et al.*, 1996; Sato *et al.*, 2009; Sun *et al.*, 2009). The glycolytic proteins 2,3-bisphosphoglycerate-dependent phosphoglycerate mutase and the 2,3-bisphosphoglycerate-independent isoform were also found to be highly affected by drought in the roots of the tested genotypes (Supplementary Table S3). Apart from playing a key role in generating energy, other functions of those enzymes were linked with fertility, vegetative growth, and defense mechanisms (Mazarei *et al.*, 2003; Zhao and Assmann, 2011). GST identified in roots showed a high level of variation among tested genotypes (Fig. 6B; Supplementary Table S3) and its accumulation pattern among RILs was found to be determined by the allele identified in Cam/B1/CI. The increase in the accumulation of GST was previously observed only in tolerant barley genotypes during osmotic stress (Witzel *et al.*, 2009). GSTs are involved in detoxification of products generated during oxidative stress, and, in particular, H_2_O_2_ (Dixon *et al.*, 2011). For example, overexpression of GST led to an increased accumulation of non-enzymatic and enzymatic components of the ascorbate–glutathione cycle and had a significant effect on neutralizing the effects of secondary oxidative stress generated during abiotic stresses (Roxas *et al*. 2000; Liu *et al.*, 2013; Sharma *et al.*, 2014). In root tissue, several enzymes involved in the phenylpropanoid pathway were also identified, which displayed a genotype-specific response, but mostly a negative drought effect (Fig. 6B; Supplementary Table S3), including phenylalanine ammonia lyase and caffeic acid *O*-methyltransferase. In our previous studies, both enzymes were also found at decreased levels in root tissue, but only in Cam/B1/CI (Chmielewska *et al.*, 2016). In plants subjected to water limiting conditions, the diminished activity of the phenylpropanoid pathway enzymes resulted in reduced content of lignins, which in turn affected the structure of the tissue (Vincent, 2005). Also, reduced biosynthesis of lignins was linked to higher drought-resistance in Arabidopsis *pal* knock-out mutants (Rohde *et al.*, 2004; Huang *et al.*, 2010).

### Large-scale proteomic data may enrich genetic linkage analysis

The studied RIL population was earlier used for linkage analysis of yield-related traits under both natural and greenhouse conditions. The localized QTLs specific to water scarcity conditions affected number of grains in the main spike, grain weight per plant, grain weight in the lateral spike and number of productive tillers (Mikołajczak *et al.*, 2016, 2017). It is noteworthy that Cam/B1/CI alleles in some cases determined positive effects for these traits under early-drought conditions. The concept of integrating proteomic data into QTL analysis has already been shown by linking the accumulation of phosphoglycerate mutase with genetic variation in drought-stressed maize cultivars (de Vienne *et al.*, 1999, 2001). Although barley genome annotation has been recently improved, very often the annotated gene sequences are described as ‘uncharacterized’. However, for several proteins, the identified pQTLs were positioned in close vicinity to the respective coding sequence; their effects could be attributed to the polymorphism at structural or *cis*-regulatory loci, as opposed to other pQTLs that are close to *trans* regulators (Supplementary Tables S7, S8). The extrapolation of data from linkage analysis to our proteomic results showed co-localization of QTLs related to yield components (Table 1). The identified pQTLs were mostly found in the regions affecting multiple phenotypic traits and, especially, on chromosome 3H and 5H. In gene expression profiling studies, the major expression QTLs associated with drought were found also on those chromosomes (Wehner *et al.*, 2016). The semi-dwarfing *sdw1/denso* locus (found in Maresi) is one of the main determinants of plant height, and many other yield-related traits in barley co-segregate with the region identified in the 3H.1 linkage group (Kuczynska and Wyka, 2011; Mikołajczak *et al.*, 2017). In the same region, two enzymes implicated in the phenylpropanoid pathway and lignin biosynthesis were also mapped: PAL isoforms from leaves and roots and caffeic acid *O*-methyltransferase from roots (Supplementary Tables S7, S8). In gibberellin-deficient dwarf plants, the activity of PAL was found to be a limiting factor of lignification, suppression of which was linked with increased tolerance to drought (Cheng and Marsh, 1968; Macarisin *et al.*, 2009; Huang *et al.*, 2010). Also, in the reported metabolic QTL analysis of the studied RILs, one of the drought-responsive phenylpropanoid pathway products was mapped to the same SNP marker as the root PAL (Piasecka *et al.*, 2017). Proteomic QTLs for root betaine-aldehyde dehydrogenase and P5CS, which are involved in glycine betaine and proline biosynthesis, respectively, were also positioned together with QTLs related to several yield-related traits (Table 1; Supplementary Table S8). Recently, sequence variations in the P5CS gene were associated with drought tolerance and yield-related traits in barley (Xia *et al.*, 2017). Also, in the studied RILs, osmolytes constituted a group of metabolites the accumulation of which was significantly increased under drought conditions (Swarcewicz *et al.*, 2017). In many plant species, the elevated levels of osmolytes were correlated with higher yield and increased plant growth under various abiotic stresses (Ashraf and Foolad, 2007; Chmielewska *et al.*, 2016). The pQTLs for BiP and thioredoxin in leaves co-localized with QTLs for grain weight per plant, heading stage, and length of main spike, which further suggests the importance of defense mechanisms for yield generation during abiotic stresses (Table 1; Supplementary Table S7; Chmielewska *et al.*, 2016). The increasing effect of the Cam/B1/CI allele on pQTL for the β-subunit of ATP synthase in leaves also positively affected QTLs determining length of main stem and main spike, number of grains, grain weight per lateral spike and heading stage in drought and control conditions, which links energy metabolism and yield capacity (Table 1; Supplementary Table S7; Mikołajczak *et al.*, 2017).

## Conclusions

Our approach to the analysis of a large number of 2D gels proved to be a reliable method and similar results were obtained with regard to earlier drought-related proteomic studies. However, in future research, new strategies for protein identification based on LC-MS/MS methods should be applied since they require less workload than 2D gel electrophoresis and provide more detailed data (Niu *et al.*, 2018). The results generated in our study enabled the enrichment of the genetic linkage map of the studied RILs for additional proteomic levels. The biological functions of the identified drought-responsive proteins have already been linked with various mechanisms involved in drought adaptation. However, unlike previously, the statistical evaluation of large-scale proteomic data facilitated the discrimination between proteins that responded to drought to the same extent among tested genotypes and proteins for which the change in accumulation additionally strongly fluctuated among barley genotypes, for example Rubisco activase B (no. 140) and luminal binding proteins (nos 86 and 87) in leaves, and phosphoglycerate mutase (nos 10 and 177), small HSP (no. 125), GST (no. 114), phenylalanine ammonia-lyase (no. 169), and caffeic acid *O*-methyltransferase (no. 59) in roots. The specific protein isoforms exhibiting strong G×E interactions may constitute the potential targets for further research on drought resistance. The incorporation of pQTLs into biotechnologically driven marker-assisted breeding programs may constitute an additional tool for selecting plants with desired agronomic traits and result in novel varieties with increased drought resistance in shorter time.

## Data deposition

The script performing the matching procedure, written in Genstat 17, together with the 2D gel images and input data files generated by IMP7 were deposited in the Dryad Digital Repository. https://doi.org/10.5061/dryad.6st3v7r.

## Supplementary data

Supplementary data are available at *JXB* online.

Fig. S1. Workflow of the experiments in the project.

Fig. S2. Gel alignment procedure.

Table S1. The minimum information about a proteomics experiment (MIAPE)—gel electrophoresis.

Table S2. Leaf proteins observed in RILs—the results of statistical analysis and mass spectrometry identification.

Table S3. Root proteins observed in RILs—the results of statistical analysis and mass spectrometry identification.

Table S4. Differences between protein levels in leaves and drought effects observed in parental genotypes: Maresi and Cam/B1/CI.

Table S5. Differences between protein levels in roots and drought effects observed in parental genotypes: Maresi and Cam/B1/CI.

Table S6. Protein classification by differences between parental genotypes: Maresi and Cam/B1/CI.

Table S7. Proteomic quantitative trait loci identified for leaf proteins.

Table S8. Proteomic quantitative trait loci identified for root proteins.

Supplementary Figures S1-S2 Tables S1 S6

Supplementary Table S2

Supplementary Table S3

Supplementary Table S4

Supplementary Table S5

Supplementary Table S7

Supplementary Table S8
