# Supplementary material for: Identification of drought responsive proteins and related proteomic QTLs in barley
Source: J Exp Bot. 2019 Feb 28;70(10):2823–37. doi: 10.1093/jxb/erz075 (PMC6506773; doi:10.1093/jxb/erz075)
Supplement: Supplementary Figures S1-S2 Tables S1 S6 [file erz075_suppl_supplementary_figures_s1-s2_tables_s1_s6.pdf]

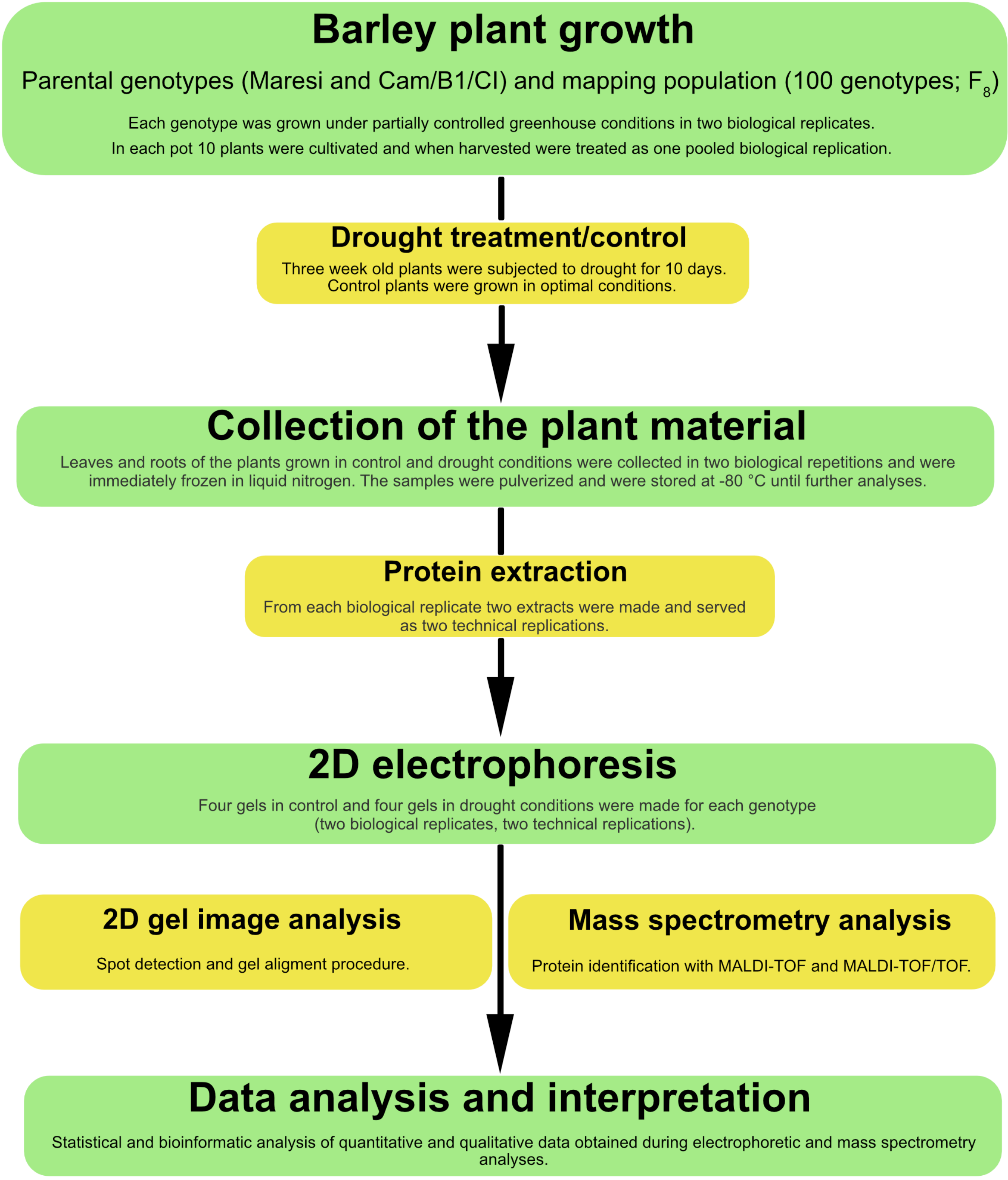

Figure S1. Workflow of the experiments in the project. The segregating population of 100 RILs of spring barley (*Hordeum vulgare* L.), and their parental genotypes – Maresi and Cam/B1/CI were used for the experiments. To ensure high level of homozygosity, F8 plants were used for the analyses.

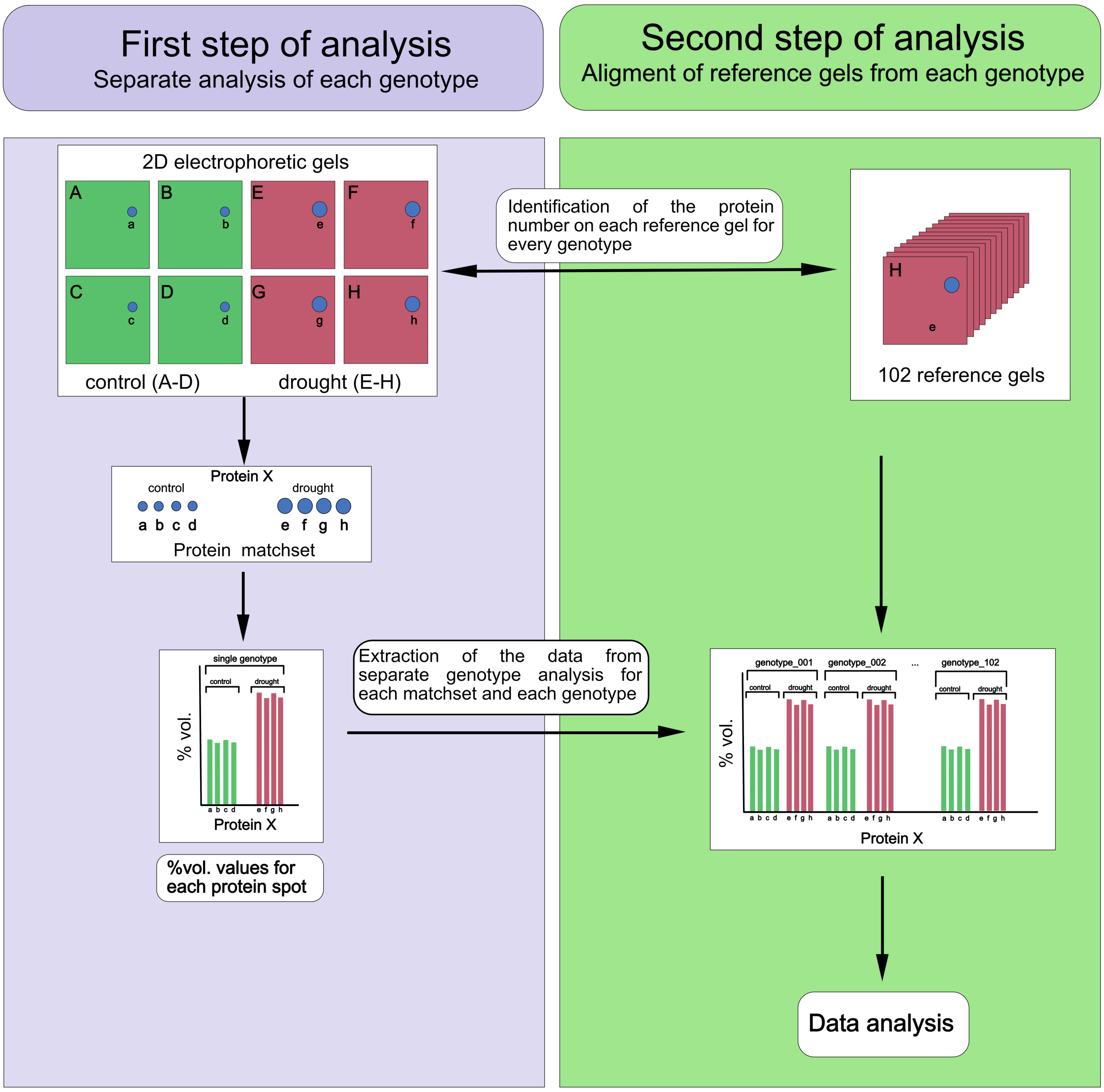

Figure S2. Gel alignment procedure. We developed a two-step matching procedure allowing to identify spots in all gels. In the first step, gels obtained from control and drought plants (in 2 biological and 2 technical replications) were matched and annotated for each genotype separately in IMP7 software. Subsequently, 102 reference gels (for each genotype - first replication under drought) were matched in IMP7. Then, the protein spot numbers on reference gels were used to match sets of spots obtained for different genotypes. In other words, protein spots found in gels for different genotypes were matched via their identifiers in reference gels. The observations of %vol. for each measured protein spot from all genotypes and replications were extracted to a joint result file.

Table S1. The minimum information about a proteomics experiment (MIAPE) - gel electrophoresis

Classification	*Definition*
1. General features	
1.1.1 Date stamp	20140101
1.1.2 Responsible person or role	**Prof. Maciej Stobiecki**<br>Institute of Bioorganic Chemistry PAS, Department of Biochemistry of Natural Products, Noskowskiego 12/14, 61-704 Poznań, Poland, Tel.+48 61 852 85 03, e-mail: mackis@ibch.poznan.pl
1.1.3 Electrophoresis type	Two-dimensional gel electrophoresis
2. Sample	
2.1.1 Sample name(s)	**Sample names**:<br><br>**Parent genotypes:**<br>[parent genotype]_[condition]_[biological replicate]_[technical repetition]_[tissue]<br><br>**RILs:**<br>RIL_[RIL number]_[condition]_[biological replicate]_[technical repetition]_[tissue]<br><br>**Parent genotypes:** Maresi, Cam/B1/CI<br>**RIL number:** 001, 002, 004, 005, 006, 007, 008, 009, 010, 014, 017, 019, 021, 024, 026, 027, 028, 029, 035, 039, 041, 042, 043, 044, 045, 046, 048, 049, 050, 052, 053, 056, 057, 058, 059, 060, 061, 063, 065, 066, 067, 068, 069, 070, 071, 072, 073, 074, 075, 076, 077, 078, 079, 080, 081, 082, 084, 085, 086, 087, 088, 089, 090, 091, 092, 093, 094, 096, 097, 099, 0100, 0101, 102, 103, 104, 105,

Table S6. Protein classification by differences between parental genotypes

Leaves

Protein function	Drought effect in					
	Maresi			Cam/B1/CI		
	Negative	No effect	Positive	Negative	No effect	Positive
Carbon metabolism	10	22	2	1	33	2
Defence	12	13	8	6	25	5
Gene expression	3	5	1	2	8	0
Ion/electron transport	2	8	3	1	9	3
Nitrogen metabolism	6	7	0	2	11	0
Other	3	3	2	0	6	2
Photosynthesis	12	23	7	4	34	7
Secondary metabolism	0	0	2	0	2	0
Signaling	0	1	0	0	1	0
Structural	0	0	1	0	2	0
Unknown	6	41	6	8	48	10
Total	54	123	32	24	179	29
Total in variety*		209			232	

Roots

Protein function	Drought effect in					
	Maresi			Cam/B1/CI		
	Negative	No effect	Positive	Negative	No effect	Positive
Carbon metabolism	5	30	7	5	29	9
Defence	8	61	11	11	77	12
Gene expression	1	9	3	2	12	1
Ion/electron transport	1	11	2	1	12	3
Nitrogen metabolism	7	13	2	2	15	0
Other	1	8	0	1	8	0
Secondary metabolism	3	7	0	5	5	0
Unknown	7	102	12	7	119	14
Total	33	241	37	34	277	39
Total in variety*		311			350	

Protein level	Leaf	
	Control	Drought
Higher in Maresi	24	21
Equal	154	159
Higher in Cam/B1/CI	20	41
Total number of proteins*	198	221

Protein level	Root	
	Control	Drought
Higher in Maresi	33	52
Equal	247	279
Higher in Cam/B1/CI	16	13
Total number of proteins*	296	344

* Less than 257 (leaves) and 381 (roots) as some proteins were not observed in parental lines

	106, 107, 108, 109, 110, 111, 112, 113, 114, 115, 116, 118, 119, 120, 121, 122, 123, 124, 125, 126, 127, 128, 129, 130<br>**Condition:** control, drought<br>**Biological replicate:** 1, 2<br>**Technical repetition:** 1, 2<br>**Tissue:** leaf, root
2.1.2 Loading buffer	**Rehydration buffer:**<br>7 M urea, 2 M thiourea, 2% (w/v) CHAPS
3. Gel matrix and electrophoresis protocol – 3.1. Dimension details	
3.1.1 Ordinal number for this dimension	**First**<br>**Second**
3.1.2 Separation method employed	**First** - isoelectric focusing<br>**Second** – SDS-PAGE
3. Gel matrix and electrophoresis protocol – 3.2. Gel matrix	
3.2.1 Description of gel matrix	**First** – IPG strips, gradient pH 4-7<br>**Second** - 12% SDS-polyacrylamide gels
3.2.2 Gel manufacture	**IPG strips** – GE Healthcare (USA)<br>**SDS-polyacrylamide gels** – prepared “in house”
3.2.3 Physical dimensions	**IPG strips** – 11 cm<br>**SDS-polyacrylamide gels** – 11 cm x 11 cm

**Ingredient**	**Final concentration**
Acrylamide/N,N'-Methylenebisacrylamide (40%)	12%
Tris-HCl, pH 8.8 (1.5M)	40 mM
TEMED	0.07% (v/v)
SDS (10%)	0.1% (v/v)
Ammonium persulfate (10%)	0.05% (v/v)

<table>
<tr><td>3.2.4 Physicochemical property range and distribution</td><td>IPG strips – linear pH gradient
SDS-polyacrylamide gel – resolution 100-10 kDa</td></tr>
<tr><td>3.2.5 Acrylamide concentration</td><td>SDS-polyacrylamide gel – 12%</td></tr>
<tr><td>3.2.6 Acrylamide:Crosslinker ratio</td><td>Acrylamide: N,N'-Methylenebisacrylamide – 37.5 : 1</td></tr>
<tr><td>3.2.7 Additional substances in gel</td><td>Not applicable</td></tr>
<tr><td>3.2.8 Gel lane</td><td>Not applicable</td></tr>
<tr><td>3.2.9 Sample application</td><td>All samples (2.1.1) were processed in the same manner. The samples containing 300 µg of proteins were loaded onto IPG strip (rehydration loading).

Volume of loading buffer – 300 µl</td></tr>
<tr><td colspan="2">3. Gel matrix and electrophoresis – 3.3. Protocol</td></tr>
<tr><td>3.3.1 Buffer</td><td>Anode buffer – Tris (25 mM)
Catode buffer – Tris (25mM), glycine (200 mM), SDS (0.1% w/v)</td></tr>
<tr><td>3.3.2 Electrophoresis conditions</td><td>Isoelectric focusing:
Temperature: 19°C
<table>
<tr><th>Mode profile</th><th>Voltage</th><th>Time [h:min]</th><th>kVh</th></tr>
<tr><td>-</td><td>0</td><td>3:00</td><td>0</td></tr>
<tr><td>Step and hold</td><td>50</td><td>9:00</td><td>0.45</td></tr>
<tr><td>Gradient</td><td>50-500</td><td>0:40</td><td>0.25</td></tr>
<tr><td>Step and hold</td><td>500</td><td>1:00</td><td>0.5</td></tr>
<tr><td>Gradient</td><td>500-1000</td><td>0:20</td><td>0.25</td></tr>
<tr><td>Gradient</td><td>1000-6000</td><td>2:30</td><td>8.8</td></tr>
<tr><td>Step and hold</td><td>6000</td><td>2:30</td><td>15</td></tr>
<tr><td>SUM</td><td></td><td>19</td><td>24.8</td></tr>
</table></td></tr>
</table>

<table>
<tr><td></td><td>SDS-PAGE:
Temperature – 19°C
Wattage – 1.25 W per gel (30 min); 7.5 W per gel (2 h)</td></tr>
<tr><td colspan="2">4. Inter-dimension Process – 4.1 Protocol</td></tr>
<tr><td>4.1.1 Step name</td><td>Reduction and alkylation</td></tr>
<tr><td>4.1.2 Inter-dimension buffer</td><td>Reduction buffer
<table>
<tr><th>Ingredient</th><th>Final concentration</th></tr>
<tr><td>Urea</td><td>6 M</td></tr>
<tr><td>Glycerol</td><td>29% (v/v)</td></tr>
<tr><td>Tris-HCl, pH 8.8</td><td>75 mM</td></tr>
<tr><td>SDS</td><td>2% (w/v)</td></tr>
<tr><td>Bromophenol blue 1%</td><td>0.002% (v/v)</td></tr>
<tr><td>Dithiotreitol</td><td>1% (w/v)</td></tr>
</table>
Alkylation buffer
<table>
<tr><th>Ingredient</th><th>Final concentration</th></tr>
<tr><td>Urea</td><td>6 M</td></tr>
<tr><td>Glycerol</td><td>29% (v/v)</td></tr>
<tr><td>Tris-HCl, pH 8.8</td><td>75 mM</td></tr>
<tr><td>SDS</td><td>2% (w/v)</td></tr>
<tr><td>Bromophenol blue 1%</td><td>0.002% (v/v)</td></tr>
<tr><td>Iodoacetamide</td><td>2.5% (w/v)</td></tr>
</table></td></tr>
<tr><td>4.1.3 Additional reagents</td><td>Not applicable</td></tr>
<tr><td>4.1.4 Equipment</td><td>Isoelectric focusing was performed using Ettan IPGphor 3 System (GE Healthcare, USA).
SDS-PAGE was performed using Ettan DALTtwelve System (GE Healthcare, USA).</td></tr>
<tr><td>4.1.5 Protocol</td><td>Reduction:
Protein samples loaded onto IPG strips were reduced for 15 min in 7.5 ml of reduction buffer.</td></tr>
</table>

	**Alkylation:**<br>Protein samples loaded onto IPG strips were alkylated for 15 min in 7.5 ml of alkylation buffer.
5. Detection – 5.1 Direct detection	
5.1.1 Name of direct detection process	**Visible staining**
5.1.2 Direct detection agents	**Coomassie Brilliant Blue (colloidal solution)**<br><br>**Ingredient** – **Final concentration**<br>Coomassie Brilliant Blue (G-250) – 0.1% (w/v)<br>Ammonium sulfate – 10% (w/v)<br>Phosphoric acid (85%) – 2% (w/v)
5. Detection – 5.2 Indirect detection	
5.2.1 Name of indirect detection process	**Not applicable**
5.2.2 Transfer medium	**Not applicable**
5.2.3 Detection medium	**Not applicable**
5.2.4 Indirect detection agents	**Not applicable**
5.2.5 Additional reagents and buffers	**Not applicable**
5.2.6 Equipment	**Not applicable**
5.2.7 Indirect detection protocol	**Not applicable**
6. Image Acquisition – 6.1. Acquisition Equipment	
6.1.1 Type of equipment	**Optical scanner**
6.1.2 Name of equipment	**Image Scanner III** (GE Healthcare, USA)
6.1.3 Software	**LabScan 6.0** (GE Healthcare, USA)
6.1.4 Calibration	**Paper Gray Scale** (KODAK, USA);

	density range 0-1.8, 12 density increments, step width 20 mm
6.1.5 Equipment specific parameters	**Not applicable**
6. Image Acquisition – 6.2. Acquisition Protocol	
6.2.1 Image acquisition process	**Transparent mode scanning**
6.2.2 Reference to gel matrix	**Polyacrylamide gel** (section 3)
7. Image	
7.1.1 Image name	**The image files refer to sample names (section 2.1.1)**<br>**File extension:** *.mel*<br>e.g. RIL_001_control_1_1_leaf.mel
7.1.2 Dimensions	1200px x 1200px
7.1.3 Resolution	300 dpi
7.1.4 Bit-depth	16 bit
7.1.5 Image location	Dryad Digital Repository. https://doi.org/10.5061/dryad.6st3v7r
7.1.6 Standard image orientation	**Molecular mass:** high (top), low (bottom);<br>**pH gradient:** pH 4 (left), pH 7 (right)
